# Discovery of Novel Hypermethylated Genes in Prostate Cancer Using Genomic CpG Island Microarrays

**DOI:** 10.1371/journal.pone.0004830

**Published:** 2009-03-13

**Authors:** Ken Kron, Vaijayanti Pethe, Laurent Briollais, Bekim Sadikovic, Hilmi Ozcelik, Alia Sunderji, Vasundara Venkateswaran, Jehonathan Pinthus, Neil Fleshner, Theodorus van der Kwast, Bharati Bapat

**Affiliations:** 1 Samuel Lunenfeld Research Institute, Mount Sinai Hospital, Toronto, Ontario, Canada; 2 Department of Laboratory Medicine and Pathobiology, University of Toronto, Toronto, Ontario, Canada; 3 Department of Pediatric Laboratory Medicine, the Hospital for Sick Children, Toronto, Ontario, Canada; 4 Division of Urology, Sunnybrook Health Sciences Centre, Toronto, Ontario, Canada; 5 Department of Surgery, Division of Urology, McMaster University, Hamilton, Ontario, Canada; 6 Division of Urology, Department of Surgery, University Health Network, University of Toronto, Toronto, Ontario, Canada; 7 Department of Pathology, University Health Network, University of Toronto, Toronto, Ontario, Canada; 8 Department of Pathology and Laboratory Medicine, Mount Sinai Hospital, Toronto, Ontario, Canada; Ordway Research Institute, United States of America

## Abstract

**Background:**

Promoter and 5′ end methylation regulation of tumour suppressor genes is a common feature of many cancers. Such occurrences often lead to the silencing of these key genes and thus they may contribute to the development of cancer, including prostate cancer.

**Methodology/Principal Findings:**

In order to identify methylation changes in prostate cancer, we performed a genome-wide analysis of DNA methylation using Agilent human CpG island arrays. Using computational and gene-specific validation approaches we have identified a large number of potential epigenetic biomarkers of prostate cancer. Further validation of candidate genes on a separate cohort of low and high grade prostate cancers by quantitative MethyLight analysis has allowed us to confirm DNA hypermethylation of *HOXD3* and *BMP7*, two genes that may play a role in the development of high grade tumours. We also show that promoter hypermethylation is responsible for downregulated expression of these genes in the DU-145 PCa cell line.

**Conclusions/Significance:**

This study identifies novel epigenetic biomarkers of prostate cancer and prostate cancer progression, and provides a global assessment of DNA methylation in prostate cancer.

## Introduction

Prostate cancer (PCa) is the most commonly diagnosed cancer in men and the second leading cause of cancer associated with deaths in the US [1]. Studies have shown that PCa is a complex disease impacted by genetic and non-genetic epidemiological factors, and early diagnosis is critical in the clinical management of the disease. A common pathological variable given during of the prostate tumour, with higher scores reflecting poorly differentiated carcinoma. Gleason score ≤6 carcinomas are considered low grade, Gleason 7 is intermediate grade, and those with Gleason score 8 and above are regarded as high grade (for recent review on grading system , see [3]).

Epigenetic modifications have been shown to affect gene expression patterns and often contribute to the pathogenesis of many cancers [4]. Examples of epigenetic histone modifications include methylation of specific lysine residues, acetylation/deacetylation of lysine residues, and phosphorylation of histone tails, each having varying effects on the regulation of gene transcription. These modifications induce abnormal gene expression patterns and thus are considered to contribute to cancer development [5], [6]. Aberrant CpG dinucleotide methylation is a well recognized epigenetic hallmark of many cancers. Global genomic hypomethylation is found in conjunction with localized regions of hypermethylation, typically in CpG islands that commonly occur in the promoters or 5′ regions of gene sequences [7]. Promoter hypermethylation acts together with specific histone modifications to silence genes by direct inhibition of transcription factor biding [8], through binding of methyl CpG binding domain proteins [9], or through interactions with histone modifying enzymes [10]. This epigenetic mechanism can confer a growth advantage to cancer cells by hypermethylation of tumour suppressor genes. Accordingly, DNA methylation events may serve as useful biomarkers [11], propelling a search for both diagnostic and prognostic indicators.

CpG island hypermethylation in PCa is a common event with over 30 hypermethylated loci currently identified [12]. The best characterized of these events, *GSTP1* promoter methylation, occurs in >90% of cancers and 70% of precursor high grade prostatic intraepithelial neoplasia (PIN) lesions [13], [14] and can also be detected in blood and urine samples [15]. Thus, *GSTP1* methylation may serve as a useful diagnostic marker for PCa. Recently, substantial progress has been made in the high-throughput epigenomic screening for the identification of novel targets of DNA methylation [16]. Subsequently, other well characterized hypermethylated genes have been identified in PCa including *RASSF1A*, *CDH1*, and *CDKN2A*, to name a few. However, no gene studied to date has been identified as a specific diagnostic/prognostic biomarker in PCa similar to *GSTP1* [17], [18].

In this study, we sought to analyze methylation on a genome wide scale using human CpG island microarrays to uncover novel methylatled loci within prostate cancer. Among a panel of novel and/or differentially methylated loci that we identified, we further characterized *HOXD3* and *BMP7* using a combination of MassARRAY® EpiTYPER analysis and quantitative MethyLight assay, and assessed expression in DU-145 PCa cells.

## Methods

### Patient Samples

20 fresh frozen PCa tissue samples (10 Gleason score 6 or pure pattern 3 (PP3), and 10 Gleason score 8 or pure pattern 4 (PP4)) obtained from prostatectomy specimens of patients with prostate cancer diagnosed between 2001 and 2007 were collected from the tissue bank at the University Health Network (UHN), Toronto. Patients who had therapy prior to surgery were excluded. Another series of specimens consisting of 39 formalin-fixed, paraffin-embedded (FFPE) PCa samples (20 PP3 and 19 PP4) from patients diagnosed between 2006 and 2008 were similarly collected for the validation set. All patients consented to the donation of removed tissue to the UHN tissue bank and samples were obtained according to protocols approved by the Research Ethics Board from Mount Sinai Hospital (MSH) and UHN, Toronto, ON, Canada. PCa specimens were subjected to histological examination by an expert pathologist (TVDK) for independent confirmation of the Gleason grades.

### Cell lines and DNA extraction

Human PCa cell lines LNCaP (ATCC # CRL- 1740), DU-145 (ATCC # HTB-81), PC-3 (ATCC # 59500) and 22RV1 (ATCC # CRL- 2505) were obtained from Drs. M. Zielinska, R. Bristow, and E. Diamandis. All cells were cultured as monolayers in RPMI 1640 media (Life Technologies), and supplemented with 10% fetal bovine serum. All cell lines were grown in humidified atmosphere with 5% CO_2_ at 37°C. DNA was extracted after harvesting the cells by trypsinization followed by DNA extraction using QIAamp DNA mini kit (Qiagen Inc, Mississauga, ON, Canada), using the protocol recommended by the supplier.

### 5-Aza 2′ –deoxycitidine (DAC) treatment and RT-PCR

A 250 µg/ml stock solution of 5- aza- 2-deoxycitidine (DAC) (Sigma-Aldrich, Oakville, ON, Canada) was prepared in water and kept at −80°C until use. DU-145 cells were plated in 6 cm dishes and incubated in culture medium with 2 µg/ml DAC for 4 days with medium change every 2 days. Cells were harvested and total RNA was extracted using Trizol (Invitrogen, Carlsbad, CA), using the protocol recommended by the supplier.

Primer sequences for RT-PCR of *BMP7* and *HOXD3* have been described previously [19], [20] and are as follows: (*BMP7* forward) 5′-AGA GCA TCA ACC CCA AGT-3′, (*BMP7* reverse) 5′-CTA CTC AGG CCC CAG CTT-3′; (*HOXD3* forward) 5′-AGG ATC CTG GTC TGA ACT CAG AGC AGC AGC3′, (*HOXD3* reverse) 5′-ACT CGA GTT CAT CCG CCG GTT CTG GAA CCA-3′.

### DNA isolation

Fresh frozen archived tissue was snap-frozen in liquid nitrogen, crushed, and genomic DNA was isolated using the QIAamp DNA mini kit (Qiagen) according to the kit protocol. FFPE tissue was sectioned (7 µm) and air-dried onto slides. Areas with a distinct Gleason grade in H&E stained slides with at least 80% or more neoplastic cells were marked and the corresponding areas were identified on FFPE sections for harvesting cells. Separate specimens with histologically confirmed normal tissue were marked as well. The enriched cell populations from highlighted areas were then manually microdissected and genomic DNA was isolated using the QIAamp DNA mini kit using a modified protocol with extended proteinase K digestion. Briefly, microdissected tissue was digested in 30 µL proteinase K at 56°C overnight, followed by an addition of 20 µL proteinase K and digestion for one hour at 56°C the following day. The Qiagen recommended protocol for FFPE tissue was then followed.

### Differential Methylation Hybridization (DMH) and Human CpG Island Microarrays

The differential methylation hybridization technique for preparation of methylated amplicons was carried out as described previously [21]. Briefly, genomic DNA (0.2 µg) from PP3 and PP4 cases was digested with *Mse*I. The cleaved ends were ligated with annealed H-12/H-24 linkers, followed by further digestion with two successive rounds of digestion with methylation-sensitive enzymes, namely *Hpa*II and *Bst*UI. Linker PCR reactions were then performed with pre-treated DNA to generate the final target amplicons for microarray hybridization. Final amplicons were purified using the QIAquick PCR purification kit (Qiagen) according to the manufacturer's protocol. The reference sample consisted of DNA isolated from lymphocytes of six healthy men age-matched with PCa patients. Reference samples were similarly treated for final target generation and pooled amplicons were co-hybridized to the test cases for individual arrays.

### Data Analysis

All microarray data generated is compliant with current MIAME standards according to Brazma *et al* [22].

Statistical analyses were performed with the statistical package ***limma*** of *R* [23]. The principle is to fit a linear model for each probe where the log_2_ ratio of red channel intensity and green channel intensity is regressed on a tumour indicator variable (I). We performed three comparisons: Pure Pattern 3, Gleason 6 (PP3) (I = 1) vs. Reference (I = 0), Pure Pattern 4, Gleason 8 (PP4) (I = 1) vs. Reference (I = 0), and PP4 (I = 1) vs. PP3 (I = 0), to find genes that have different methylation profiles across the two groups compared. These comparisons are analogous to a classical two-sample *t*-test analysis. Alternatively, we also used an empirical Bayes *t*-test. This has the same interpretation as an ordinary *t*-statistic except that the standard errors have been moderated across genes (shrunk towards a common value) using a simple Bayesian model. This has the effect of borrowing information from the ensemble of genes to make the inference about each individual gene more robust. The moderated *t*-statistic has an increased number of degrees of freedom compared to the ordinary *t*-statistic, reflecting the greater reliability associated with the smoothed standard error. Our analyses were conducted after pre-processing the data. In the first case, we used a background correction method provided by Agilent. In the second case, we used a method implanted in ***limma***. A convolution of normal and exponential distributions is fitted to the foreground intensities using the background intensities as a covariate, and the expected signal given the observed foreground becomes the corrected intensity. This results in a smooth monotonic transformation of the background subtracted intensities such that all the corrected intensities are positive. Both methods performed well on our data. We then applied a loess normalization procedure within arrays to remove any systematic trends which arise from the microarray technology from the methylation measures [24].

### Partek Data Analysis and Integration

Data from Agilent Feature Extraction software .txt were analysed using the Partek Genomic Suite Software (PGS) using a modification of the previously described protocols [25], [26]. The processed R and G column data from 10 PP3 and 10 PP4 were imported into PGS. The processed R signal corresponded to the tumour DNA and processed G signal corresponded to the normal lymphocyte DNA. The cancer-specific signal across all probes was normalized as a ratio to baseline using Normalize to Baseline Tool in PGS, where baseline data corresponded to normal human lymphocyte DNA. The data was then log_2_ transformed using the PGS Normalization and Scaling Tool.

Such normalized and transformed dataset was then used for detection of cancer specific methylation profiles, and secondarily to differentiate between PP4 and PP3-specific methylation profiles. In order to detect significant cancer-specific enrichment/depletion, we performed Hidden Markov Model (HMM) region detection across approximately 244,000 probes with the following parameters: minimum probes: 5, detection states: −2 & 2; ignore state: 0, maximum probability: 0.95, genomic decay: 10,000, sigma: 1. Such detected genomic regions were annotated to the corresponding genes using the PGS gene annotation tool with Affymetrix HuGene-1_0-st-v1.na24.hg18.transcript.csv file. In addition to the directly overlapping genes, proximal genes (up and downstream 1000 nucleotides) to the enriched/depleted regions were also annotated.

Significant differences in enrichment between PP3 and PP4 tumours were identified by calculating the average fold difference between the PP3 and PP4 normalized signal across all probes using the PGS ANOVA tool, and subsequent HMM region detection and gene annotation using the above mentioned parameters. Such genomic regions were further filtered to include sequences with minimum 1.3 fold enrichment, and minimum −1.3 fold depletion. The visualization of data using heat maps, .wig files for UCSC Genome Browser, genome view files, and corresponding data tables/lists was performed using PGS as previously described [25], [26].

### MassARRAY EpiTYPER Analysis

Quantitative analysis of CpG dinucleotide methylation was performed using a mass spectrometry approach as available by MassARRAY® EpiTYPER analysis (Sequenom). EpiTYPER analysis is a MALDI TOF mass spectrometry based method that provides a quantitative view of CpG dinucleotide methylation to single or multiple dinucleotide resolution. DNA is first bisulfite modified, tagged with a T7 promoter, and transcribed into RNA. This is then cleaved with RNase A and cleavage products of different mass can be resolved by the MS instrument. Analysis was performed by the Analytical Genetics Technology Centre (AGTC), Princess Margaret Hospital, Toronto, ON as per manufacturer's instructions using a subset of fresh frozen tissue DNA that was used for CpG island microarray analysis. Regions analyzed by EpiTYPER corresponded to those that showed an enriched signal in the CpG island array results. All analyses were performed in triplicate and averages and standard errors were calculated.

### Sodium Bisulfite Modification and MethyLight

Sodium bisulfite modification of genomic DNA was carried out using the EZ DNA Methylation Gold Kit (Zymo Research Corp, Orange, CA, USA) according to the manufacturer's protocol using 0.8 µg of paraffin- embedded tissue DNA.

Methylation levels of the two genes of interest were determined by quantiative methylation specific PCR (MSP), the MethyLight assay, as described previously [27]. Primers and probes were designed specifically for bisulfite converted, methylated DNA and are as follows: (*BMP7* forward) 5′-CGT TTT TTT GGT TCG GAT CGC-3′, (*BMP7* probe) 6FAM-5′- GTG TCG AGA GGG TAG GGT CGG TTT CG-3′-BHQ1, (*BMP7* reverse) 5′-CTA AAA CCT AAC GAA ACG TCG CG-3′; (*HOXD3* forward) 5′- GTT TTG GTA TTT CGG GTT TTT ATC G-3′, (*HOXD3* probe) 6FAM-5′- AAG AGC GTT TGG GGG AGG GGG GC-3′-BHQ1, (*HOXD3* reverse) 5′-TAA AAC TCC TAA CTT CGC GCT ACG-3′; (*Alu* forward) 5′-GGT TAG GTA TAG TGG TTT ATA TTT GTA ATT TTA GTA-3, (*Alu* probe) 6FAM-5′-CCT ACC TTA ACC TCC C-3′-MGBNFQ, (*Alu* reverse) 5′-ATT AAC TAA ACT AAT CTT AAA CTC CTA ACC TCA-3′. All reactions were performed on the Applied Biosystems 7500 Real Time PCR instrument. Standard curves were generated using serial dilutions of positive control supermethylated DNA for the gene of interest and *Alu* repeats. Percent methylated ratio (PMR) for a gene was calculated using *Alu* repeats as reference as follows: (gene/*Alu* fluorescence quantity ratio for modified specimen DNA) / (gene/*Alu* ratio for supermethylated DNA) X 100%. A positive score for methylation was given if PMR for a given tumour was ≥10%.

## Results

### Analysis of genomic methylation

We separated the analysis of our microarray data into two subsets. The first subset consisted of all 20 cancer specimens compared to reference DNA. A list of genes that were identified as significantly hypermethylated in the statistical methods performed for the cancer versus reference dataset (PP3&PP4 versus reference DNA) is depicted in Table 1. Interestingly, 27 of the top 100 methylated genes (ranked by individual probe fold change) from the cancer/reference dataset are homeobox or T-box genes (Table 2), consistent with current literature analyzing methylation patterns in other cancers including those of the lung, breast, and colon [28], [29], [30]. We also found >2 fold signal in genes previously identified as methylated in prostate cancer such as *CDKN2A* (average of 15.8 fold enrichment), *RUNX3* (2.8 fold), and *PTGS2* (2.9 fold). The gene showing the greatest degree of methylation was *FOXC1* with an average fold change of 60.9 versus the reference DNA. Using PGS, which restricted analysis to multiple probes showing enrichment, the greatest degree of methylation in a characterized gene was *HOXD9* (3.2 fold change across 8 probes).

**Table 1:** Representative genes and average PGS fold change (across multiple probes) from the top 100 for cancer/reference and progression dataset.

Cancer/Reference Dataset		Progression Data Set	
Gene Name (abbreviation)	Fold Change	Gene Name (abbreviation)	Fold Change
Chromosome 20 open reading frame 103 (*C20orf103*)	3.7	Ventral anterior homeobox 1 (*VAX1*)	2.7
Homeobox D9 (*HOXD9*)	3.2	Homeobox D3 (*HOXD3*)	2.3
Nuclear receptor subfamily, group A, member 2 (*NR5A2*)	3.1	CAP-GLY domain containing linker protein family (*CLIP4*)	2.1
Distal-less homeobox 5 (*DLX5*)	3.1	Calcium channel, voltage dependent, T-type, alpha 1G subunit (*CACNA1G*)	2.0
Iroquois homeobox 1 (*IRX1*)	3.0	Glycoprotein V (*GP5*)	1.9
Spastic paraplegia 20 (*SPG20*)	3.0	Somatostatin receptor 1 (*SSTR1*)	1.8
Transcription factor AP-2 alpha (*TFAP2A*)	2.9	Methylthioadenosine phosporylase (*MTAP*)	1.8
Wilms tumor 1 (*WT1*)	2.9	NK2 homeobox 2 (*NKX2-2*)	1.7
SIX homeobox 6 (*SIX6*)	2.8	Homeobox C11 (*HOXC11*)	1.6
Homeobox D4 (*HOXD4*)	2.7	Ladybird homeobox 1 (*LBX1*)	1.6
Transcription factor 7-like 1 (*TCF7L1*)	2.6	Motor neuron and pancreas homeobox 1 (*MNX1*)	1.6
Sonic hedgehog homolog (*SHH*)	2.5	Glutamate receptor, metabotropic 1 (*GRM1*)	1.6
Protocadherin, gamma subfamily C,5 (*PCDHG5*)	2.4	LIM homeobox 9 (*LHX9*)	1.6
Methionine aminopeptidase 1D (*MAP1D*)	2.3	Microtubule-associated protein tau (*MAPT*)	1.5
Runt-related transcription factor 1 (*RUNX1*)	2.3	Galactosidase, beta 1-like (*GLB1L*)	1.5

**Table 2:** Representative homeobox genes showing methylation for cancer/reference and progression dataset (genes in bold overlap with Table 1).

Cancer/Reference Dataset		Progression Data Set	
Gene Abbreviation	Gene Name	Gene Abbreviation	Gene Name
*FOXC1*	Forkhead box C1	***VAX1***	**Ventral anterior homeobox 1**
***SIX6***	**Six homeobox 6**	***HOXD3***	**Homeobox D3**
*HHEX*	Hematopoietically expressed homeobox	*TBX15*	T-box 15
***HOXD9***	**Homeobox D9**	*GSC*	Goosecoid homeobox
*HOXC13*	Homeobox C13	*PROX1*	prospero homeobox 1
*TBX4*	T-box4	*TBX3*	T-box 3
*HOXD8*	Homeobox D8	*PAX2*	Paired box 2
*IRX6*	Iroquois homeobox 6	*ALX4*	Aristaless-like homeobox 4
*BARX2*	BARX homeobox 2	*PHOX2A*	Paired-like homeobox 2a
*DLX6*	Distal-less homeobox 6	*HOXD8*	Homeobox D8

The second subset of data compared the ten PP3 cases to the 10 PP4 cases, which we termed the progression dataset. Using a 2-fold average enrichment signal difference between the two patterns as a cut-off, we discovered a set of 493 array probes that are able to distinguish between PP3 and PP4 cancers. We then filtered out multiple probes representing the same gene and probes representing uncharacterized locations, giving a final list of 223 individual genes. One specific probe representing the CAP-GLY domain containing linker protein family, member 4 (*CLIP4*) showed the greatest fold difference between the two patterns (6.5). Using PGS for statistical analysis, ventral anterior homeobox 1 (*VAX1*) displayed the greatest average fold difference (2.7) over multiple probes (6 total). A representative view of genes from PGS analysis is given in Table 1. Similar to the cancer/reference dataset, 23 of the top 100 genes ranked by probe fold change from the progression dataset are homeobox genes (Table 2).

We next selected two genes from these lists for further analysis using a combination of methylation and expression based techniques. Selection criteria included the biological function of the gene, involvement in /contribution to prostate cancer, and statistical significance from CpG microarray results.

### Gene specific methylation analysis

The genes chosen for analysis were:

1. *BMP7* [Bone Morphogenic Protein] (chromosome # 8p21), a gene already implicated in PCa progression [31] which was previously reported as methylated in an oligodendroglioma cell line and gastric cancers [32], [33]. We decided to further investigate its methylation profile because of its putative downregulation in PCa progression [31] and observed methylation signal in our cancer/reference dataset (3.2 fold enrichment), suggesting that methylation of this gene may play a role in PCa progression.
2. *HOXD3* [Homoebox transcription factor] (chromosome # 2q31-37), a gene found to be methylated in lung cancer cell lines and primary tumours [34], which showed a distinct pattern of increasing methylation with tumour grade in our series based on average enrichment difference (6.4), suggesting that methylation of this gene may be involved in disease progression as well.

**Figure 1:**
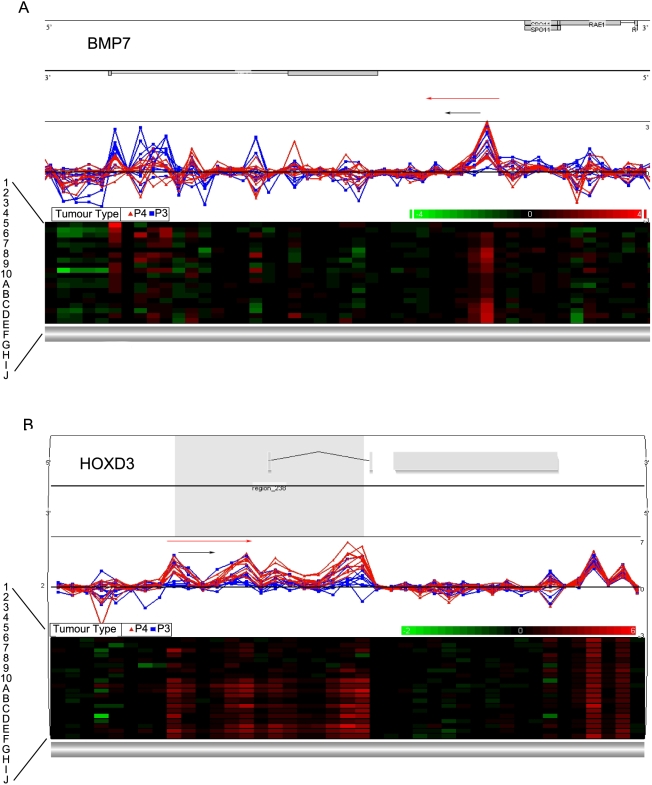
Partek Genomics Suite Visualization. (A) *BMP7* and (B) *HOXD3*. Line graphs in the upper panel of each show log_2_ ratio values for each probe, with red representing PP4 cases A–J and blue representing PP3 cases 1–10. The lower panel of each is a heat map for each probe in individual PP4 and PP3 cases. Red arrows correspond to regions selected for EpiTYPER analysis while black arrows correspond to regions chosen for MethyLight analysis.

Partek graphical and heatmap visualization of the microarray data is shown for the two genes in figure 1.

### EpiTYPER quantitation of CpG Methylation

**Figure 2:**
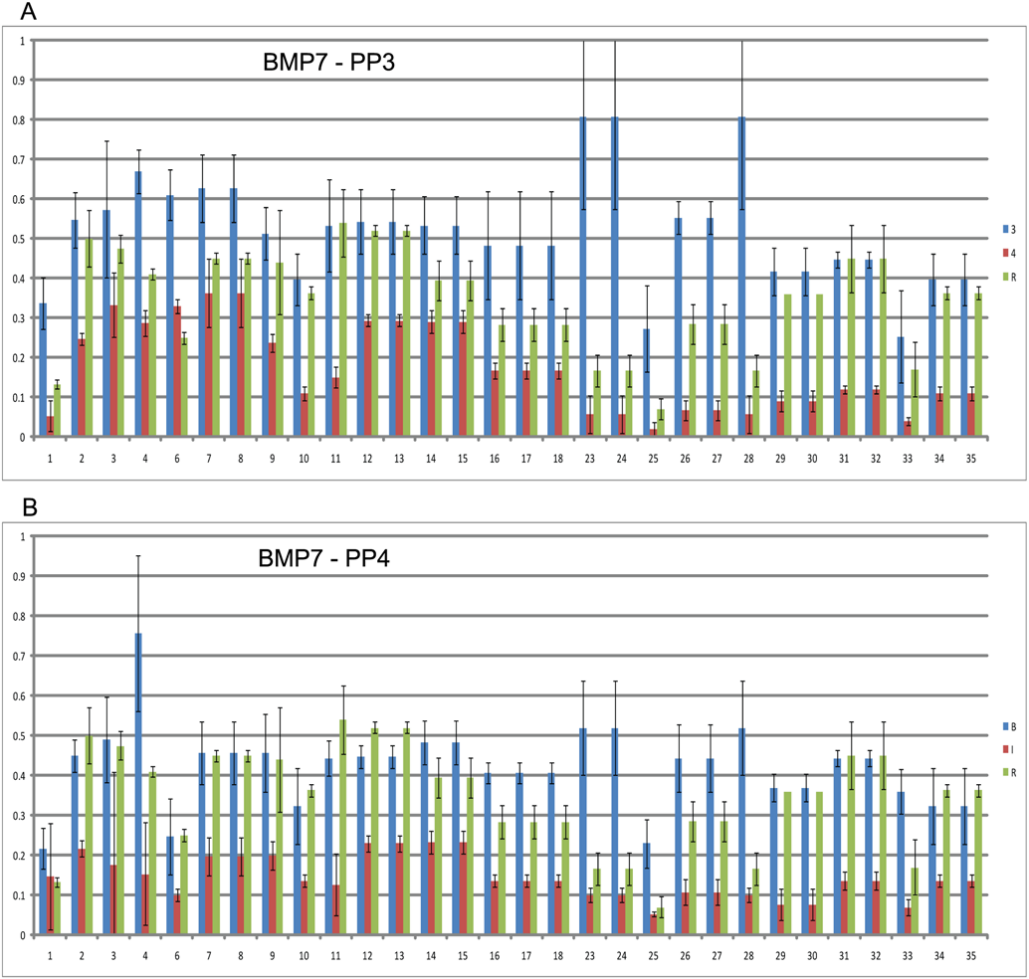
EpiTYPER analysis of *BMP7*. (A) PP3 cases 3 and 4 and (B) PP4 cases B and I. Reference lymphocyte is shown for each. Coloured bars represent the average methylation over three replicates with standard error bars displayed.

**Figure 3:**
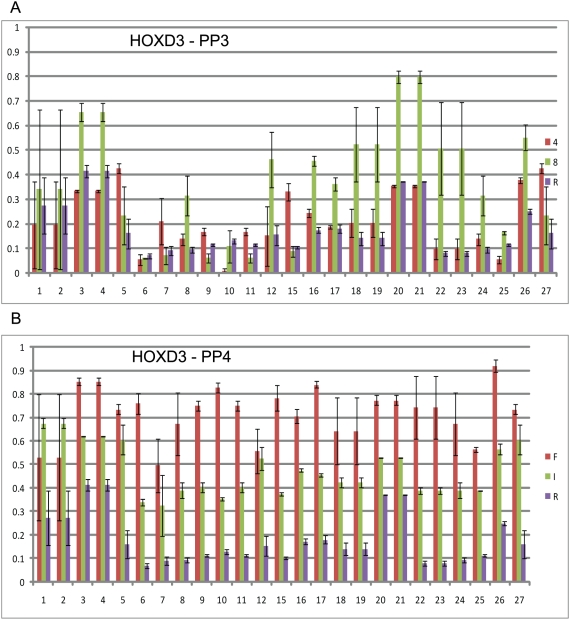
EpiTYPER analysis of *HOXD3*. (A) PP3 cases 4 and 8 and (B) PP4 cases F and I. Reference lymphocyte is shown for each. Coloured bars represent the average methylation over three replicates with standard error bars displayed.

The EpiTYPER analysis included a subset of cases that showed enrichment of ≥3 fold or a lack of methylation signal (≤2 fold) on the microarrays. Data obtained from EpiTYPER analysis confirmed the enrichment/methylation profiles in *BMP7* and *HOXD3* that were evident from the microarray results in a set of four microarray cases chosen for analysis (Figures 2, 3). For *BMP7*, methylation of the region identified by our microarray analysis confirmed that for samples B and 3, there was a significant level of methylation compared to that of the reference DNA (up to 76% for CpG dinucleotide 4 in sample B) (figures 2A,B). These samples had an average methylation of 43% and 52% (methylated/unmethylated ratio, given as percent), respectively, across all 35 CpGs analyzed, while samples I and 4 showed an average CpG methylation of 14% and 17%, respectively. *HOXD3* displayed a distinct pattern of increased methylation in the PP4 cases as compared to the PP3 cases. The analysis of fresh frozen DNA samples F, I, 4, and 8 confirmed a differential pattern of methylation from PP3 to PP4, at least with respect to the four cases analyzed (figure 3A, B). High grade cases F and I had an average methylation of 72% and 43% respectively, across all 27 CpG dinucleotides analyzed, while low grade samples 4 and 8 respectively had an average methylation of 19% and 35%.

### MethyLight Analysis

**Figure 4:**
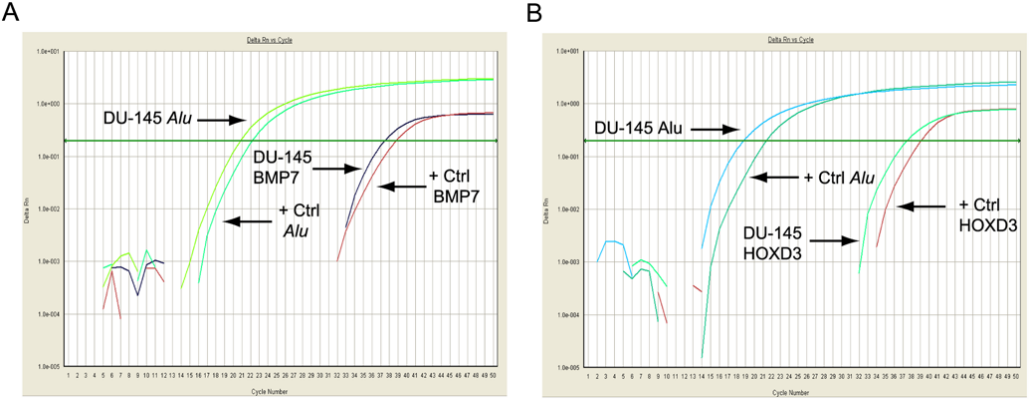
Amplification plots for MethyLight analysis of DU-145 cells. (A) *BMP7* and (B) *HOXD3* MethyLight amplification plots. The x-axis shows the cycle number while the y-axis shows the delta Rn value. +Ctrl – supermethylated DNA.

To verify methylation patterns of these genes, we validated them in an independent series of paraffin embedded PCa cases, with matched normal tissue from the same specimens where available, and also assessed their methylation status in PCa cell lines (DU-145, PC-3, 22RV1, and LNCaP) using MethyLight. *BMP7* methylation was verified in a total of 4 tumour specimens (two PP3, two PP4) as well as two normal samples from separate cases (figure 4A). *HOXD3* methylation was present in a total of eight specimens (two PP3, six PP4) (figure 4B). DU-145 cells were positive for methylation of both *BMP7* and *HOXD3*. PMR values for DU-145 and positive cases are given in table 3.

**Table 3:** PMR values of positive cases for *BMP7* and *HOXD3* methylation.

*HOXD3*	PMR(%)	*BMP7*	PMR(%)
DU-145	61	DU-145	110
PP3		PP3	
i	16.8	iii	11
ii	50	iv	19.6
PP4		PP4	
a	16	g	15
b	30	h	11.2
c	80		
d	81		
e	80		
f	17		

### RT-PCR

**Figure 5:**
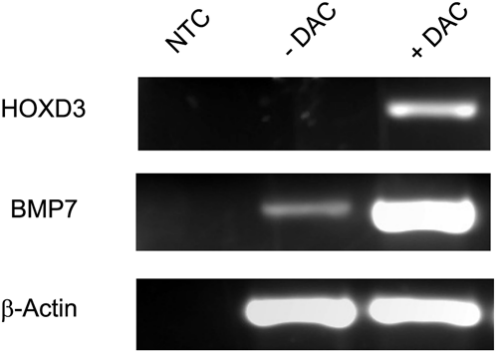
RT-PCR analysis of DU-145 cells. NTC – no template control. −DAC – untreated. +DAC – treated with 5-aza-2-deoxycytidine.

We next treated DU-145 cells with the demethylating agent DAC and performed semi-quantitative RT-PCR analysis using untreated and treated cells to assess the effect of methylation on expression on the two genes. *HOXD3* expression appears to be completely abolished in untreated DU-145 cells while *BMP7* is minimally expressed. Treatment with DAC induced *HOXD3* expression and caused an increase in *BMP7* mRNA levels (Figure 5), indicating that methylation is involved in the reduced expression of both *BMP7* and *HOXD3*.

## Discussion

We have used human CpG island microarrays to identify methylated genes in PCa as a whole, as well as differentially methylated in low grade, PP3 and high grade, PP4 PCa. Intermediate Gleason score 7 tumours were not examined as they are composed of both patterns 3 and 4, and we chose to narrow our focus to those tumours that are composed entirely of Gleason pattern 3 or Gleason pattern 4 in order to have enriched cell pattern populations. We found that we were able to identify CpG islands that are both quantitatively more methylated and methylated at an increased frequency in PP4 tumours when compared to PP3 tumours. This may reflect an overall shift to a greater state of methylation within promoter CpG islands as the tumour progresses towards a higher grade.

Most genes uncovered through our arrays have either never been shown to be methylated in PCa or in other types of cancers. Other previously described methylated genes in prostate cancer, such as *CDKN2A*, *PTGS2*, and *RUNX3*, all showed evidence of methylation based on fold changes and statistical significance. The stringency of the statistical analyses that we performed could have prevented the inclusion of these genes within our top genes of the progression or cancer/reference dataset. Therefore, this may not be indicative of a lack of methylation, but instead can be explained by quantitative methylation levels. It is possible that methylation of these genes may have occurred in fewer cells and/or in a fewer number of CpG dinucleotides, thus producing a less robust signal in our screen. Alternatively, grouping of cases in statistical analyses may have filtered these genes out, since methylation in a fewer number of specimens would create a lower average and higher variability across these cases. We were surprised to find that the best characterized methylation event in PCa, hypermethylation of the *GSTP1* promoter, was not captured in our array screen results. It is possible that the method we used for target DNA preparation in combination with the microarray platform is responsible for the lack of detection of *GSTP1* methylation signal. Sequence analysis of *GSTP1* revealed that our methylated DNA enrichment method would produce a fragment of approximately 1900 bp, which may affect annealing to probes of significantly smaller length (approximately 45–60 mer) or may not remain intact following methylation sensitive digestion. Upon further investigation of *GSTP1* methylation in the same 20 cancer specimens, however, we could detect methylation in 80% of cases using MSP (data not shown).

We developed a list of genes comparing the total cancer dataset versus reference, as well as separating methylation profiles for PP3 and PP4 Gleason scores. We chose to do an in-depth methylation analysis of *BMP7* and *HOXD3*, as these are novel targets for methylation in PCa. They represent a subset of genes where silencing may play a role in the development of high grade prostate cancers based on our array results, but also based on available functional information from current literature. Therefore, these genes do not necessarily reflect the greatest statistical significance or the greatest methylation fold change of either two datasets that we produced. The genes with the greatest fold changes in the datasets, *FOXC1* and *VAX1*, will require future validation in a larger series of prostate tumours.

Bone morphogenic proteins are secreted factors that control the development and maintenance of bone formation and belong to the TGFβ superfamily of signalling proteins [35]. Within PCa it has been shown that *BMP7* is significantly underexpressed in laser microdissected cancer cells, leading to an epithelial-to-mesenchymal transition [31]. It does, however, appear to be re-expressed in metastatic PCa foci of the bone [36]. Our discovery of promoter methylation in *BMP7* suggests a possible mechanism through which the initial silencing is achieved, as treatment of DU-145 cell lines with DAC increased *BMP7* expression dramatically. Previous studies have shown methylation of *BMP7* in gastric cancers and oligodendroglioma cell lines [32], [33], suggesting that silencing of *BMP7* through this mechanism is not limited to PCa alone. Of note, *BMP7* methylation was not exclusive to histologically cancerous tissue, but was also evident in adjacent normal tissue. This may be ascribed to the field cancerization effect whereby methylation occurs prior to any histological cancerous change in the cells, which has been shown to occur in prostate cancer [37]. Alternatively, this methylation may be primarily age-related, as this phenomenon has also been shown before in normal prostate tissue [38]. Future studies are required to address these issues.

*HOXD3*, another novel PCa methylation target, showed a distinct shift towards greater levels of methylation from PP3 to PP4 PCa when analyzing our CpG array results. The role that *HOXD3* plays in tumourigenesis and/or progression of the disease has yet to be identified, but activation of TGFβ signalling has been shown in A549 cells transfected with *HOXD3* [39]. Aberrations in this pathway have been well documented in PCa and other cancers [40]. It is therefore possible that methylation-induced silencing of *HOXD3* is perturbing TGFβ signalling, and perhaps contributing to the development of high grade PCa. Studies using the lung cancer cell line A549 [41] and two melanoma cell lines (A375M, MMIV) [42] suggest that overexpression of *HOXD3* leads to increased motility and invasiveness in these cancers, and is not expressed in normal melanocytes. The overall difference in methylation captured by our CpG array screen was recapitulated by analyzing a separate set of PCa samples, from which we could detect a modest increase in promoter methylation between the PP3 and PP4 cases (2 vs. 6, respectively) using MethyLight. In addition, we found a quantitative difference between PP3 and PP4 with EpiTYPER analysis. This difference may represent an overall increase in neoplastic cells with hypermethylated *HOXD3* promoters, contributing to an overall pattern of high Gleason grade. It is important to note that *HOXD3* is expressed at detectable levels in normal prostate [43], as many homeobox genes are regulated in a spatial and temporal manner. Using DU-145 cells, which showed exclusive methylation of the *HOXD3* promoter, we were not able to detect any expression of *HOXD3*. However, gene expression was observed following DAC treatment. Taking these two points together, it appears that aberrant methylation is responsible for abnormal silencing of *HOXD3* in PCa.

Although much of the Agilent CpG microarray covers promoter and 5′ regions of genes, it is not limited to CpG islands in and around these areas. The CpG array coverage also extends into gene bodies, downstream gene locations, and currently uncharacterized chromosomal regions. For this study, we chose to limit our validation to upstream gene promoters, as these are well characterized for their effects on silencing gene expression [44]. We did notice, however, significant methylation events occurring at all three of the aforementioned genome/chromosome locations which could have varying effects on gene transcription.

In summary, we present the discovery of two novel targets of hypermethylation in prostate cancers. We specifically chose *HOXD3* as it represents an interesting class of genes that appear to show a pattern of increased methylation correlating with tumour grade progression according to the classic Gleason pattern grading system within Gleason score 6 and 8 tumours. This pattern may be related to the aggressive biology of high grade tumours and thus deserve further investigation.
